# Supplementary material for: Membrane adsorber design for lentiviral vector recovery
Source: Mol Ther Methods Clin Dev. 2025 Jul 16;33(3):101533. doi: 10.1016/j.omtm.2025.101533 (PMC12329310; doi:10.1016/j.omtm.2025.101533)
Supplement: Document S1. Figure S1 [file mmc1.pdf]

OMTM, Volume *33*

Supplemental information

Membrane adsorber design

for lentiviral vector recovery

George Pamenter, Danyal H. Rahim, Ciaran Lamont, Maria Kapanidou, Kirstie Pemberton, Rui Sanches, Anurag Kulkarni, Oliver Goodyear, Carol Knevelman, Kyriacos Mitrophanous, Andre Krause, Florian Taft, Volkmar Thom, Daniel G. Bracewell, and Lee Davies

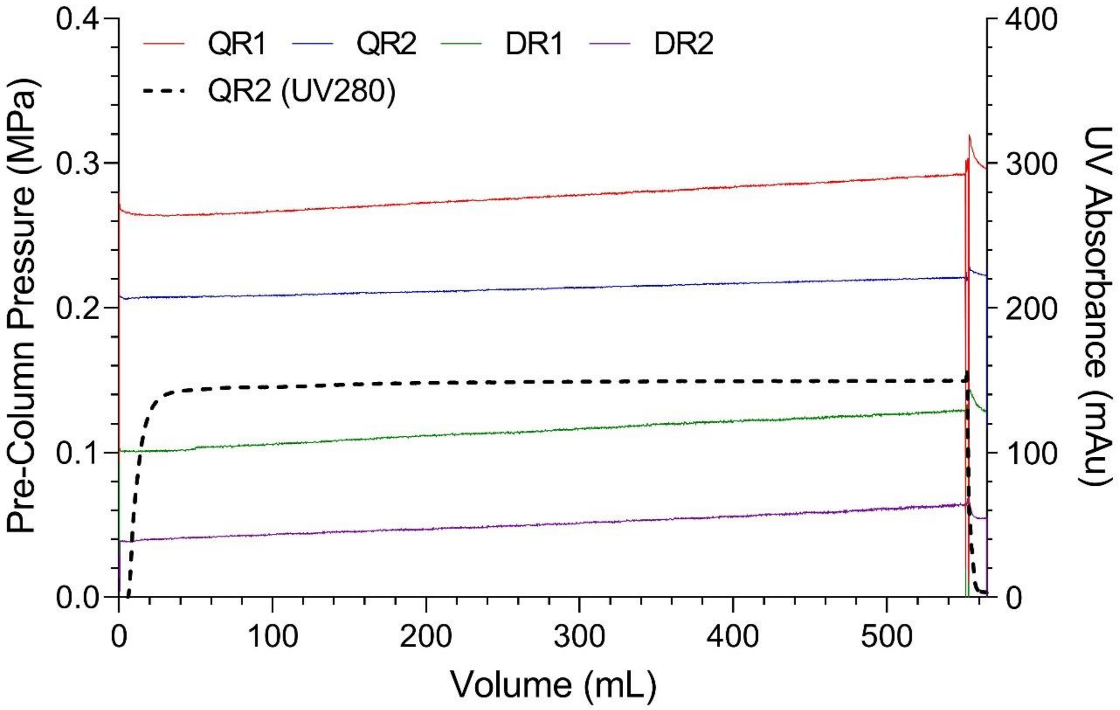

Figure S1: Pre-column pressure of the prototypes during dynamic binding capacity studies. Individual runs are given alongside the UV280 profile from a single run (QR2) to reference against for interpretation of the loading state.
