# Supplementary material for: Membrane adsorber design for lentiviral vector recovery
Source: Mol Ther Methods Clin Dev. 2025 Jul 16;33(3):101533. doi: 10.1016/j.omtm.2025.101533 (PMC12329310; doi:10.1016/j.omtm.2025.101533)
Supplement: Document S2. Article plus supplemental information [file mmc2.pdf]

Molecular Therapy
Methods & Clinical Development
Original Article

Membrane adsorber design for lentiviral vector recovery

George Pamenter,[1,2] Danyal H. Rahim,[2] Ciaran Lamont,[2] Maria Kapanidou,[2] Kirstie Pemberton,[2] Rui Sanches,[2] Anurag Kulkarni,[2] Oliver Goodyear,[2] Carol Knevelman,[2] Kyriacos Mitrophanous,[2] Andre Krause,[3] Florian Taft,[3] Volkmar Thom,[3] Daniel G. Bracewell,[1] and Lee Davies[2]

[1]Department of Biochemical Engineering, University College London, London, UK; [2]Process Science and Innovation, Oxford Biomedica (UK) Ltd., Oxford, UK; [3]PD Separation Materials, Sartorius Stedim Biotech GmbH, Göttingen, Germany

The growing demand for lentiviral vectors (LVs) in cell and gene therapies has highlighted significant challenges in large-scale LV production, particularly low product recovery. Anion-exchange chromatography (AIEX) is widely used for LV capture. However, AIEX accounts for most of the product loss in downstream processing, due to excessive interaction strengths in current commercially available membranes causing irreversible LV binding. To address this, a new AIEX membrane structure that reduces LV interaction strength by lowering ligand density, using direct ligand grafting, and incorporating tighter pore size distributions is tested. Four prototypes were created with Q and D anion-exchange chemistries at 1.05 (R1) and 1.32 (R2) μm pore sizes. Prototypes minimized product loss from irreversible binding, with high total particle recoveries irrespective of time spent in the adsorbed state (∼90% at t = 3 min, ∼80% at t = 100 min). Narrower elution ranges were shown, with LV eluted <450 mM NaCl. The best-performing prototype, DR2, exhibited a ∼3-fold higher functional product recovery than standard Q-membranes for LV encoding GFP (50%) and CAR (73%) transgenes. At large-scale, downstream processes using DR2 membranes showed a 3.5-fold improvement in functional product recovery at drug substance (43%) compared to a standard Q-membrane process (12%). These results demonstrate that adsorbents designed for lentiviral vectors significantly enhance downstream recoveries.

INTRODUCTION

As cell and gene therapy clinical trials continue to grow, a surge in the demand for high-quality viral vector products, used as vehicles to deliver genetic information to patients' cells, has also occurred.[1] Lentiviral vectors (LVs) have emerged as the leading vector for modification of patients' cells *ex vivo*, as they can achieve long-term expression of large genetic payloads (∼10 kb) in both dividing and non-dividing cells.[2–5] As such, LVs are now the established method for introducing tumor-targeting chimeric antigen receptors (CARs) to patients' T cells to produce CAR-T cell therapies used for the treatment of various cancers.[6] There are currently four Food and Drug Administration (FDA)-approved CAR-T cell therapies relying on LVs, alongside other approved LV-utilizing cell therapy products such as Zynteglo (Bluebird Bio) for the treatment of beta-thalassemia.[7] LVs are therefore utilized in a significant portion of approved cell and gene therapy products, and they also represent approximately 34% of UK clinical trials of advanced therapy medicinal products in 2023 with a disclosed vector.[8] However, the limited supply of LV continues to inhibit the progression of LV-based therapies to market. This is primarily due to challenges associated with the manufacturing process, with consistent and reliable supply of clinical-grade vector becoming a major focus of cell therapy manufacturers.

Low product recovery and poor process robustness are characteristic of LV manufacturing processes and have contributed to limited global availability of LV raw material. Third-generation LVs are derived from human immunodeficiency virus type 1 (HIV-1) and are large vectors (∽80–120 nm diameter) containing two copies of a single-stranded RNA genome encapsulated in p24 protein, which is further enveloped by a section of the host-cell membrane that enrobes the vector particle during the budding process.[9,10] This cell membrane, and thus the resulting vectors, are typically pseudotyped with the envelope "G" glycoprotein from vesicular stomatitis virus (VSV-G).[11,12] It is the structural complexity resulting from the numerous different species contained within the LV envelope environment that poses significant and unique challenges for LV bioprocessing.

LVs are routinely manufactured from transfection of human embryonic kidney (HEK) 293T cells in suspension culture and are harvested and purified from the cell culture supernatant using a bespoke combination of filtration, chromatography, and enzymatic digestion steps dependent on the vector and manufacturer.[11,12] Despite the wide variety of LV bioprocesses employed, anion-exchange chromatography (AIEX) is used ubiquitously across the industry for primary product capture due to the net negative charge of LVs at neutral pH,

Received 13 February 2025; accepted 9 July 2025;
https://doi.org/10.1016/j.omtm.2025.101533.

Correspondence: Lee Davies, Process Science and Innovation, Oxford Biomedica (UK) Ltd, Oxford, UK.
E-mail: l.davies@oxb.com

© 2025 The Author(s). Published by Elsevier Inc. on behalf of The American Society of Gene and Cell Therapy.
This is an open access article under the CC BY license (http://creativecommons.org/licenses/by/4.0/).

its broad applicability across LV pseudotypes, and its low cost. Despite this widespread application, high vector losses in the region of 70%–80% are experienced during large-scale AIEX, making it the major challenge facing cost-effective purification of LVs.[13,14] Although bead-based resins are also an option for the purification of viral vectors and have demonstrated high recoveries, they are not commonly employed for large-scale LV production due to the requirement for single-use technologies and presence of bead pores that generate diffusional mass transfer resistance and lead to dramatically reduced resin capacity for LV due to the inaccessibility of the pores.[15,16] Furthermore, resins require linear flowrates ∽10-fold lower than their membrane counterparts, which extend processing times and are not suited to LV systems due to the instability of the product and the requirement for fast processing to mitigate time-dependent loss.[17,18]

Convection-driven membrane adsorbents are therefore predominantly used for capture of viral vectors at large-scale, despite not being originally designed with this purpose in mind.[13,18–20] Current membranes are ill-suited to the complex adsorption behaviors manifesting in LV systems.[18,21] In our previous work, we demonstrated a primary loss mechanism wherein extended durations in the bound state result in irreversible binding of LV.[18] It was assumed that this was associated with strong charge interactions causing a conformational change in LV that resulted in higher degrees of multipoint attachment. We were unable to mitigate this loss mechanism by removing the strongest binding LV envelope species (glycosaminoglycans) to produce LV with weaker interaction strengths.[21] These data imply a better solution to reducing time-dependent loss may be manipulating the adsorbent design rather than LV structure.[21] As currently available AIEX membranes were not designed with viral vectors in mind, they possess design features that are ill-suited for their effective purification. For example, in Q-membrane adsorbers, elevated ligand densities have been shown to not only reduce the recovery of Adenovirus 5 (Ad5) but they also significantly impair the titer and infectivity of eluted vector.[22] A similar effect was also seen for purification of baculoviruses.[23] Furthermore, Turnbull et al. directly correlated higher ligand densities with increased time-dependent loss of Ad5 vectors on Q-nanofibers.[24] As current commercial membranes were developed to maximize performance characteristics such as capacity, and not to minimize these vector-specific loss mechanisms, it is likely that current ligand densities are too high.[22]

In commercial AIEX membranes (Sartobind Q), the Q-ligand is distributed within and on the surface of a grafted polymer (hydrogel-grafted) with a layer thickness of approximately 0.5 μm.[25] This approach is commonly used in membrane adsorbers to enhance binding capacity. For example, in Ad5 purification using Sartobind Q, removing the hydrogel resulted in over 60% of the virus being lost in the flowthrough from capacity reduction.[22] However, hydrogel grafting also promotes multi-point attachment.[26] As we have previously implicated multipoint attachment in the loss of LV product over time on AIEX Q-membranes, hydrogel grafting is likely unsuitable for LV separation.[18] Furthermore, hydrogel-grafted membranes have been associated with a failure to close the material balance in Ad5 systems, suggesting irreversible viral retention on the membrane in these vector systems also.[22] Since AIEX recovery, not binding capacity, is the primary driver of LV cost of goods, we concluded that any capacity gains from hydrogel grafting are outweighed by the risk of recovery losses.[14] Based on these considerations, this study deliberately assessed membranes without hydrogel grafting.

In this work, we ascertain whether AIEX membranes with the aforementioned undesirable characteristics removed lead to significantly enhanced purification performance when compared to a current commercially available membrane, Sartobind Q, herein referred to as a standard Q-membrane. First, we test the hypothesis that excessive membrane interaction strengths, in standard Q-membranes, cause irreversible LV binding by establishing whether time-dependent loss mechanisms are mitigated at increased membrane occupancy (bound mass). A new LV-specific membrane material is then presented with an order of magnitude lower ligand density, direct ligand grafting, and a smaller more homogeneous pore size distribution. Four prototype devices were evaluated with quaternary-amine (Q) and dimethylamine (D) chemistries at two pore size distributions, narrow (R1) and wide (R2). The binding behavior of these four prototypes was evaluated via dynamic binding capacity (DBC) and gradient elution studies. Two high-performing prototypes (DR2 and QR2) were compared against a standard Q-membrane for AIEX purification of a model GFP-encoding LV (LV-GFP), with contact time studies used to ascertain whether these new membranes reduce the time-dependent loss mechanism. The most promising prototype (DR2) is then scaled to a 3 mL bed volume radial flow capsule and compared against a current commercially available membrane for the purification of a therapeutic CAR-encoding LV product (LV-CAR) at the bench scale. Finally, this new membrane material is assembled into a large-scale radial flow device format (20 mL bed volume) for purification of 3 L of load material to produce LV drug substance. Thus, demonstrating the applicability of the new membrane to large-scale LV manufacturing processes.

RESULTS

Impact of membrane occupancy on time-dependent loss of LV to the irreversibly bound state in standard Q-membranes

Our previous work showed that the recovery of HIV-1-derived LVs using standard Q-membrane adsorbents depends on the time spent in the adsorbed state.[18] Once bound, LVs likely undergo a form of conformational change from a reversibly bound to an irreversibly bound state. We hypothesize that this is due to spreading of LV particles over the membrane surface as the strong force of attraction effectively "pulls" the vector into the adsorbent, thereby increasing the degree of multipoint attachment (Figure 1Ai). To explore whether this effect could be mitigated by increasing membrane occupancy to fill the adsorbent surface and occupy "free" ligands, thus reducing multipoint attachment and irreversible binding of the vector (Figure 1Aii), we investigated the impact of loaded LV mass and time spent in the adsorbed state on LV recovery; 40, 120, and 200

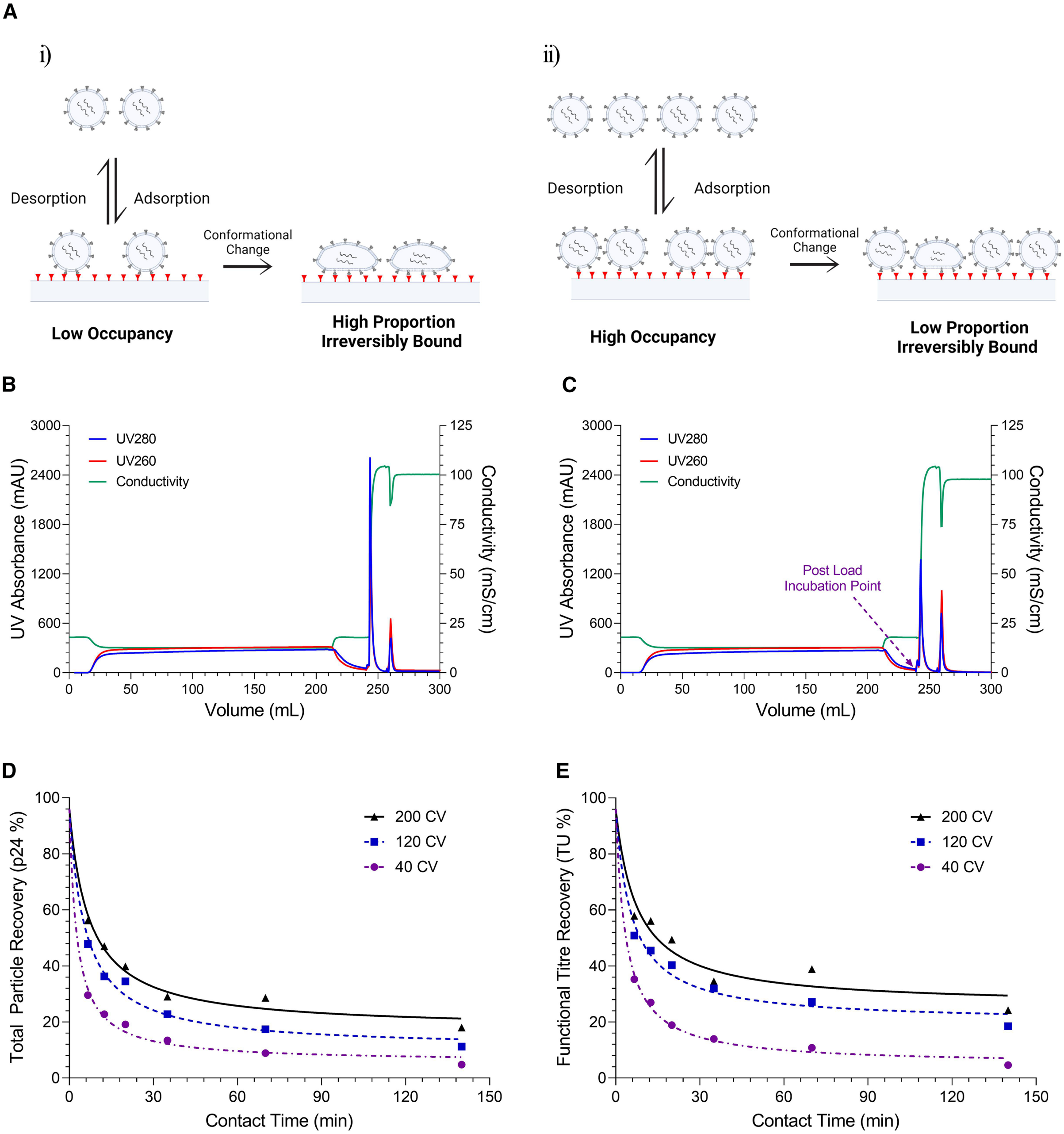

F

Load	Model Parameters	k_2(min^{-1})	Y_o	Y_∞	$t_{Y_{1/2}}$(min)	R^2
**200 CV** (92 μg_{p24}/mL)	Total Particles	1.78x10^{-3}	96	17.4	7.14	0.96
	Functional Titre	1.86x10^{-3}		25.8	7.67	0.84
**120 CV** (55 μg_{p24}/mL)	Total Particles	2.04x10^{-3}		10.5	5.74	0.96
	Functional Titre	2.29x10^{-3}		19.9	5.74	0.92
**40 CV** (18 μg_{p24}/mL)	Total Particles	4.12x10^{-3}		5.75	2.69	0.95
	Functional Titre	3.61x10^{-3}		4.81	3.61	0.98

(legend on next page)

column volumes (CV) of LV-GFP-clarified cell culture harvest (CCH) were loaded to a standard Q-membrane (representative chromatograms in Figures 1B and 1C). Kinetic profiles for LV recovery with time spent in the adsorbed state were generated by conducting a series of static "on-column" incubations, as described in Pamenter et al., for each load volume ranging from 6.61 to 140 min (example chromatogram in Figure 1C). Loss kinetics were quantified using a second order-like rate model, described in detail in Pamenter et al. and the materials and methods.[18] We previously reported non-binding studies using this feed material and determined a p24 recovery of 96%.[18] A starting recovery of 96% at t = 0 min was therefore used.

Increasing loaded volume (bound mass) led to a significant improvement in maximum total and functional particle recovery at the shortest contact time (t = 6.61 min), with recovery values increasing from 30% and 35% (40 CV) to 56% and 58% (200 CV), respectively (Figures 1D and 1E). Additionally, increasing loaded volume resulted in a longer $t_{Y_{1/2}}$ for both total and functional particles (Figure 1F), increasing from 2.69 and 3.61 min (40 CV) to 5.74 and 5.74 min (120 CV) and 7.14 and 7.69 min (200 CV), respectively. As increased membrane occupancy (bound mass) was able to mitigate vector losses, these data support the hypothesis that current membrane interaction strengths are too strong to enable effective recovery of LV. Furthermore, the high flowrates and load challenges utilized in this study make the high recoveries at t = 6.61 min practically unattainable at large scale.

It should be noted that CCH contains residual species such as DNA, host cell proteins, and other charged impurities that can bind to the membrane and occupy free ligands. Therefore, the observed reduction in time-dependent LV loss with increased membrane occupancy is likely not solely due to LV binding but also due to the binding of residuals. Consequently, cleaner material may experience greater loss due to irreversible binding.

New LV prototype membrane characterization

Based on the current hypothesis, to reduce membrane interaction strength, a new class of membranes was generated targeting LV separation. Compared to standard Q-membranes, three design elements were altered in the membrane (Figure 2A). First, overall membrane ligand density was reduced from 100 μmol/mL (standard Q-membrane) to 8 μmol/mL (prototypes). Hydrogel grafting was also removed in the prototypes, with ligands attached directly to the adsorbent surface instead. Finally, surface morphology was altered to give smaller and more homogeneous pore size. The size distribution was reduced from 3–5 μm (standard Q) to 1.05 ± 0.04 μm (R1) and 1.32 ± 0.02 μm (R2) as measured by gas-liquid porometry (data not shown). This smaller and more homogeneous surface structure was applied to maintain higher surface area, thus maintaining membrane capacity in the absence of the hydrogel. Four new membrane materials were produced using quaternary amine (Q) and dimethylamine (D) chemistries, giving QR1, DR1, QR2, and DR2 membrane prototypes.

The charged surface structure was visualized by staining each of the four prototype constructs, alongside the standard Q-membrane, with an anionic fluorescent dye (AF647 carboxylic acid) and imaging using confocal laser scanning microscopy (CLSM). Fluorescence on the standard Q-membrane is localized into high-intensity circular clusters, interspersed with areas of low to no fluorescence. In contrast, the four prototype structures exhibit a more evenly distributed, mesh-like pattern across the membrane surface due to the more homogeneous surface structure. As expected, the R2 devices display a more open structure with larger patches of no fluorescence compared to the R1 devices. These data suggest a more uniform distribution of charge across the adsorbent surface.

Dynamic binding capacity

DBC breakthrough curves, as measured by capsid p24 protein, were generated for all membranes to assess the impact of adsorbent design on LV binding capacity (Figure 3). As CCH possesses a conductivity of ∽12–14 mS/cm, we diluted it 1:1 in 20 mM Tris to achieve a loading conductivity of 7 mS/cm based on preliminary scouting studies (data not shown). All load volumes are therefore quoted as pre-dilution volumes, with membranes loaded up to 810 CV (1620 CV post-dilution). All membrane formats produced a p24 C/C_0 of approximately 0.13, likely arising from "free" p24 protein in the CCH. Soluble p24 protein has an isoelectric point of 6.7 and thus interacts very weakly with AIEX adsorbents at pH 7.2.[18] The dynamic binding capacity at 10% ($DBC_{10\%}$) for the prototype membranes was calculated to be 10% of the difference between the baseline value of 0.13 and the upper limit of 1, making its value 0.22 rather than 0.10.

The prototype membranes exhibited lower binding capacities compared to the standard Q-membrane, which had a $DBC_{10\%}$ of 76 μg p24/mL adsorbent (μg/mL_{Ad}). QR1 and QR2 had capacities of 44 μg/mL_{Ad} and 39 μg/mL_{Ad}, respectively, while DR1 and DR2 had even lower capacities of 38 μg/mL_{Ad} and 24 μg/mL_{Ad}, respectively, perhaps due to a weaker interaction strength of the D chemistry. This reduction in binding capacity is expected due to the 12.5-fold lower ligand density used in the new adsorbents. However, a ∼2-fold reduction in capacity is perhaps lower than would be

Figure 1. Impact of membrane occupancy (loaded mass) on LV-GFP product loss with time spent in the adsorbed state using standard Q-membranes
Individual runs are given. Recovery is calculated from bound material only. (A) Schematic representation of how low (i) and high (ii) membrane occupancy may impact time-dependent loss of LV on standard Q-membranes. (B) Representative chromatogram for 200 column volumes (CV) load with no incubation, giving an average time spent in the bound state of t = 6.61 min. (C) Representative chromatogram for 200 CV load with incubation point indicated in purple. The t = 140 min incubation chromatogram is given. (D) Kinetic profile of total particle recovery. (E) Kinetic profile of functional titer recovery. (F) Model fit parameters for second order-like rate equation. Y_0 was fixed to 96% for all runs as non-binding experiments for this material batch were previously demonstrated to have recoveries of 96%.[18]

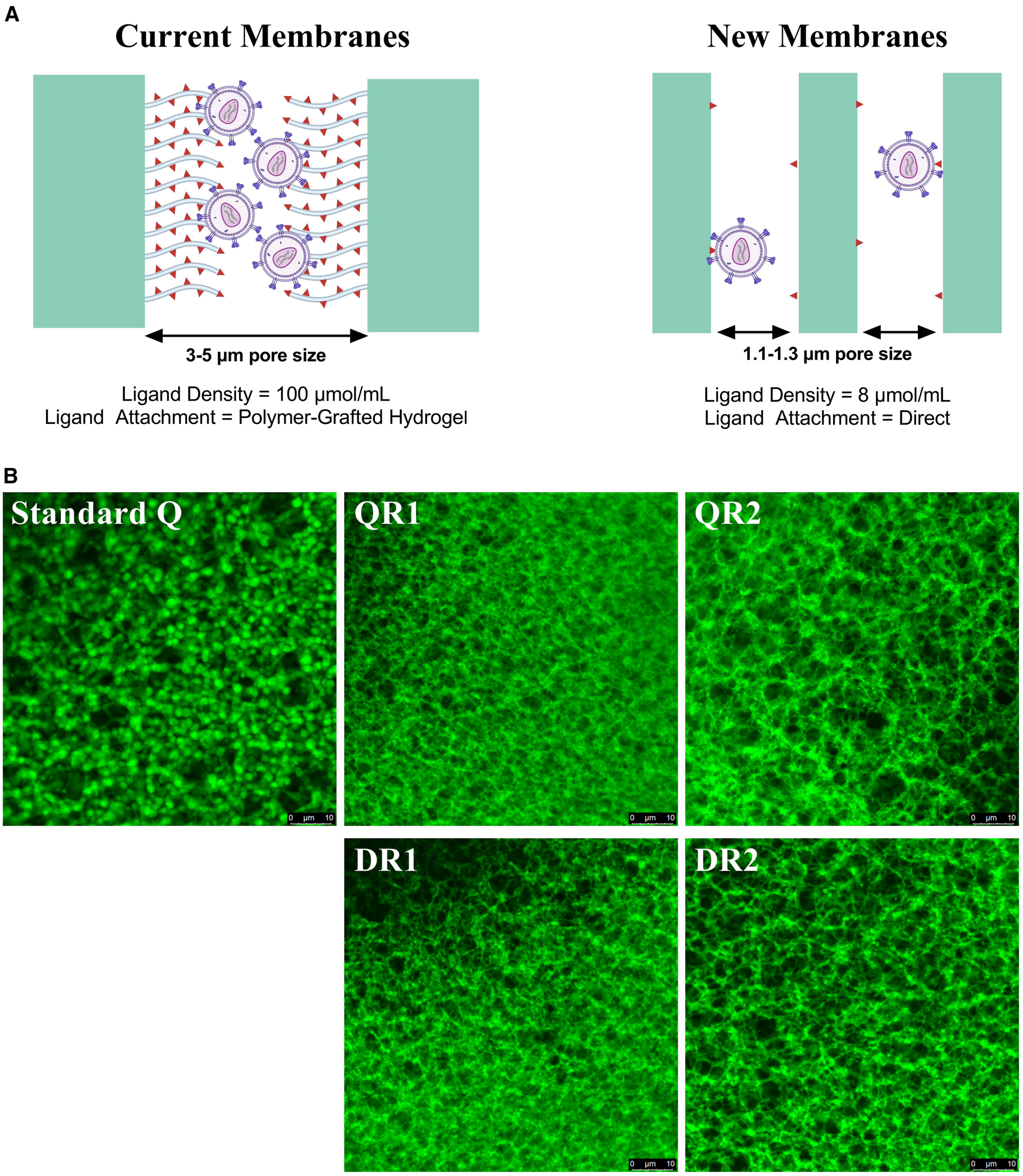

Figure 2. Difference in structure of the new prototype membranes compared to current standard Q-membrane
(A) Schematic representation of structural changes between standard Q-membranes (Current) and the prototypes (New) with ligand sites represented by red triangles and hydrogel polymer-grafting by gray tentacles. (B) Confocal laser scanning microscopy imaging of membrane surfaces upon staining with anionic fluorescent dye (AF647 carboxylic acid).

expected from this ligand density reduction and is likely the result of the optimized adsorbent surface structure increasing ligand accessibility. None of the prototypes used in this study achieved a C/C_0 value of 1.0 and showed an initial sharp rise followed by a gradual increase. In contrast, the standard Q-membrane showed a single continuous slope and plateau near $C/C_0 = 1.0$. The reasons for this are unclear but may be due to the smaller pore sizes of the prototypes causing physical retention of LV within the structure. Alternatively, more complex secondary binding interactions may cause a slower saturation after the initial slope.

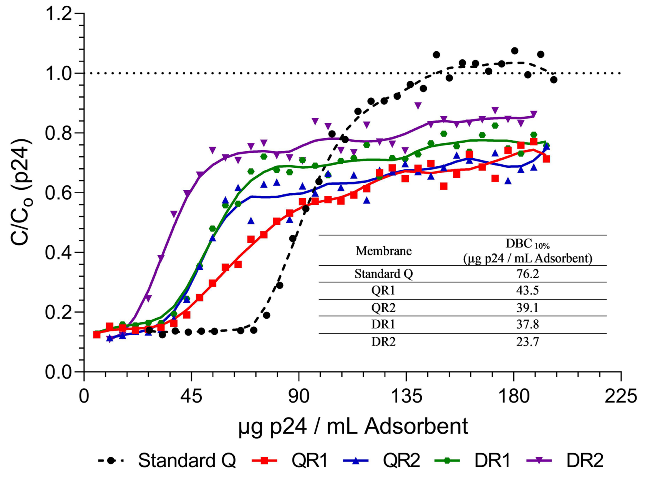

Figure 3. p24 dynamic binding capacity breakthrough curves generated using a standard Q-membrane and the four prototype membranes
Individual runs are given. $DBC_{10\%}$ values for each of the membranes are indicated on figure. As p24 not associated with vector is present in the cell culture harvest and does not bind to AIEX membranes, a breakthrough of "free" p24 is expected even at very low load challenge. Hence $DBC_{10\%}$ represents the 10% of the distance between the baseline C/Co and 1.0. The $DBC_{10\%}$ was calculated to be C/Co = 0.22.

Although analysis of membrane specific surface areas may afford deeper insight into the capacity differences observed with the prototypes, a direct comparison with standard Q membranes could not be performed. We would expect membranes with the same pore size and ligand density to have almost the same binding capacities regardless of the ligand chemistry. However, this is not the case, so the structure-capacity relationship appears more complex. A pure normalization of ligand density in relation to membrane surface could therefore be misleading. Furthermore, unlike the prototypes, the standard Q-membrane has its ligands distributed within, and on, the surface of a grafted polymer layer with a thickness of ∽0.5 μm.[25] The dynamic nature of this structure does not allow a definable viral interaction surface to be specified.

The DR1 and DR2 prototypes showed lower capacities than their Q-chemistry counterparts but had a sharper initial rise in concentration. In both chemistries, the more open pore structure (R2) resulted in lower capacity likely due to reduced specific surface area. However, R2 prototypes had significantly lower pre-column pressures at the end of loading compared to R1—QR2 (0.22 MPa) vs. QR1 (0.29 MPa) and DR2 (0.06 MPa) vs. DR1 (0.13 MPa)—consistent with their wider pore structure. The full pre-column pressure profiles for each of the prototypes is given in Figure S1. Given that capacity is of relatively low importance for LV operations, more open structures may afford greater robustness and processing speeds.

Gradient elution profiles

Gradient elution of LV from standard Q-adsorbents has typically resulted in very broad, heterogeneous elution profiles with LV eluted from 150 to 1500 mM of NaCl.[15,21,27] This is typically characterized by the presence of two peaks at low and high salt due to the presence of LV subpopulations.[21] This makes effective vector purification difficult, not only because the need to elute at high salt concentrations results in loss of vector functionality but also because broad elution ranges mean residual host cell DNA (hcDNA) and plasmid DNA (pDNA) is co-eluted with vector. The elution profiles of each prototype were evaluated against the profile from a standard Q-membrane to ascertain if differences in the typical two-peak profile were observed. For the standard Q-membrane, a gradient from 50 to 1350 mM NaCl was used. In initial scouting studies (data not shown), this gradient range was shown to be far larger than was required for the prototype membranes. Thus, to avoid excessive sampling, a shorter gradient of 50 to 750 mM NaCl was employed, but at the same gradient slope to give comparative resolution between runs.

Gradient elutions performed on standard Q-membranes loaded with diluted CCH demonstrate that LV elution begins at ∼150 mM NaCl and is followed by a heterogeneous elution consisting of two peaks at ∼300 mM and ∼900 mM NaCl (Figure 4B). Figures 4D and 4F display the elution profile of the Q- and D-functionalized prototype membranes overlayed with the standard Q-membrane for comparison. A dramatic "narrowing" of the elution profiles is observed for all prototype membranes with log higher p24 concentrations achieved at a much lower NaCl concentration and range. Most LVs are eluted before 400 mM NaCl across all prototypes, which can be attributed to the reduced virus-ligand interaction strength. For the Q-derivatized membranes, a clear two-peak profile is still observed for both QR1 and QR2, which is expected as our previous work has demonstrated this behavior stems from separate LV subpopulations present in the CCH.[21] QR2 did however result in an increase in peak spread, giving a broader elution that was slightly shifted to increased NaCl concentrations. For the D-membranes, even tighter elution peaks are observed, with most vector eluting prior to 250 mM NaCl and achieving a peak maximum of 1.39 and 1.56 μg/mL p24 for the R1 and R2, respectively. This is in stark contrast to the 1,350 mM NaCl required to elute from the standard Q-membrane, achieving a peak maximum of only 0.097 μg/mL.

In terms of UV absorbance, significantly more material is eluted from the standard Q-membrane when compared to the prototypes with peak areas of 3,435 and 2,424 for UV280 and 260, respectively (Figures 4A, 4C, and 4E). This is ∽2- to 3-fold higher than the UV absorbance of material eluted from any of the prototypes. The DR2 had the lowest residual elution, with peak areas of 931 and 974 (UV280 and 260, respectively) representing a 3.7-fold reduction and 2.5-fold reduction in UV280 and 260 when compared to the standard Q-membrane. The reduced UV elution peak area for the prototypes, compared to the standard Q-membrane, is likely driven by reduced impurity binding due to the absence of hydrogel grafting and overall lower ligand density.

Impact of membrane design on time-dependent loss of LV to the irreversibly bound state

To explore whether the membrane modifications affected the time-dependent LV loss mechanism observed in standard Q-membranes

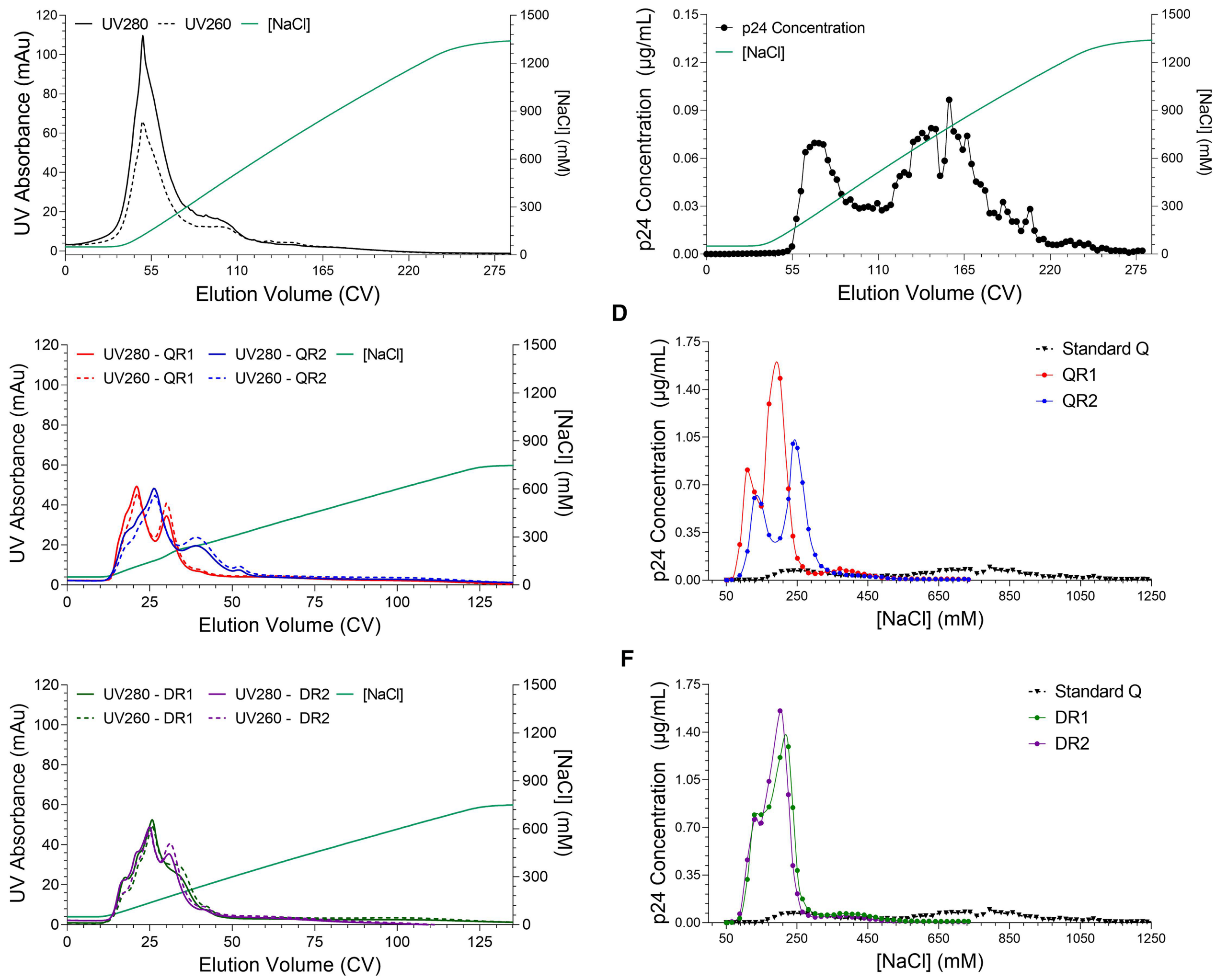

G

Eluted UV Peak Area (mAU.CV)	Standard Q	Q		D	
		R1	R2	R1	R2
UV280	3435	950	1132	999	931
UV260	2424	970	1177	1037	974
Fold Reduction (UV280)	-	3.62	3.03	3.44	3.69
Fold Reduction (UV260)	-	2.50	2.06	2.34	2.49

Figure 4. Linear gradient elution profiles for each membrane

Individual runs are given. A smoothing spline was used to generate continuous elution profiles. (A) UV280 & UV260 elution profiles of a standard Q-membrane. (B) p24 elution profile of a standard Q-membrane. (C) UV280 & UV260 elution profiles of QR1 and QR2 prototypes. (D) p24 elution profiles of QR1 and QR2 prototypes as a function of NaCl concentration. The data for a standard Q-membrane is plotted again for comparison. (E) UV280 & UV260 elution profiles of DR1 and DR2 prototypes. (F) p24 elution profiles of DR1 and DR2 prototypes as a function of NaCl concentration. The data for a standard Q-membrane is plotted again for comparison. (G) Table giving UV280 and UV260 eluted peak areas for all membranes.

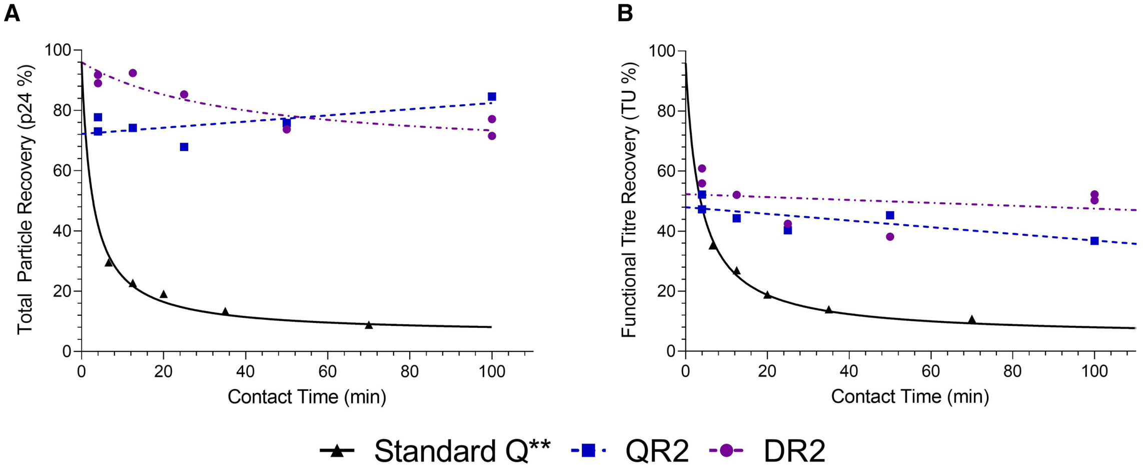

C

Membrane	Model Parameters	k_2(min^{-1})	Y_0	Y_∞	$t_{Y_{1/2}}$(min)	R^2
QR2	Total Particles	No Correlation				
	Functional Titre	No Correlation				
DR2	Total Particles	0.85x10^{-3}	96	64.8	37.7	0.84
	Functional Titre	No Correlation				
Standard-Q	Total Particles	4.12x10^{-3}	96	5.75	2.69	0.95
	Functional Titre	3.61x10^{-3}		4.81	3.61	0.98

Figure 5. Impact of membrane design on LV-GFP product loss with time spent in the adsorbed state (contact time) using the DR2 and QR2 membranes
Each replicate is given individually. (A) Impact of contact time on total particle recovery. Lines represent the model fit for the second order-like rate model. No significant correlation (p value = 0.13) could be found between total particle recovery and time for the QR2 membrane. (B) Impact of contact time on functional particle recovery. No significant correlation (p value = 0.075 for QR2, p value = 0.57 for DR2) was found for either prototype. (C) Summary of model fit parameters. **Data taken from the standard Q-membranes at 40 CV load (Figure 1) and reported here again for comparison.

(Figure 1), contact time experiments were repeated with the prototype membranes under the worst-case load volume (40 CV). As the R1 pore size demonstrated less favorable pressure rise characteristics compared to the R2 devices, in these experiments the analysis was restricted to the R2 device format only to prevent risking system overpressurization from the high flowrates required (65 CV/min).

Figure 5 demonstrates that the changes to adsorbent design had a dramatic impact on LV product loss with time spent in the adsorbed state. For the QR2 and DR2 prototypes, high total particle recoveries (Figure 5A) of 75% and 90%, respectively, were achieved at t = 4 min. Critically, for the QR2 prototype no significant correlation (p-value = 0.13) between recovery and time spent in the bound state was observed, achieving 85% recovery at t = 100 min. For the DR2 prototype, a significant correlation of time spent in the adsorbed state and total particle recovery (p value = 0.0093) was observed but the magnitude dramatically reduced when compared to standard Q membranes, with DR2 achieving a high 74% total particle recovery at t = 100 min. Applying the second order-like rate model to the DR2 data, a $t_{Y_{1/2}}$ = 37.7 min (2.69 min for standard Q) and a final recovery value of Y_∞ = 64.8% (5.75% for standard Q) are obtained.

For functional titer recovery, no significant correlation with time could be found for either QR2 or DR2 membranes, reporting a recovery value of ∽50% in both cases (Figure 5B). These data show that the new adsorbent design results in a dramatic reduction (DR2) or complete elimination (QR2) of recovery loss with time spent in the adsorbed state. This is consistent with other observations in the literature, where an inability to close mass balances for viral vectors has been observed in the presence of ligand grafting and high ligand densities.[22,24]

Step elution performance of DR2 and QR2 compared to standard Q-membranes

Having established enhanced characteristics of the new membrane structure, we now wished to see how these membranes compared against a standard Q-membrane for a comparative purification of LV-GFP. Thus, step elutions were performed in triplicate for the standard Q-membrane, QR2 and DR2 prototypes. As the aim of future work was to assess the highest performing candidates in a large-scale purification process, we restricted this analysis to R2 devices only as the manufacture of R1 membranes into full capsule devices was not possible. All CCH loads were diluted 1:1 in 0 mM NaCl Tris-based buffer and all membranes loaded with 58 CV. Standard Q-membranes were eluted with 1,200 mM NaCl and prototypes

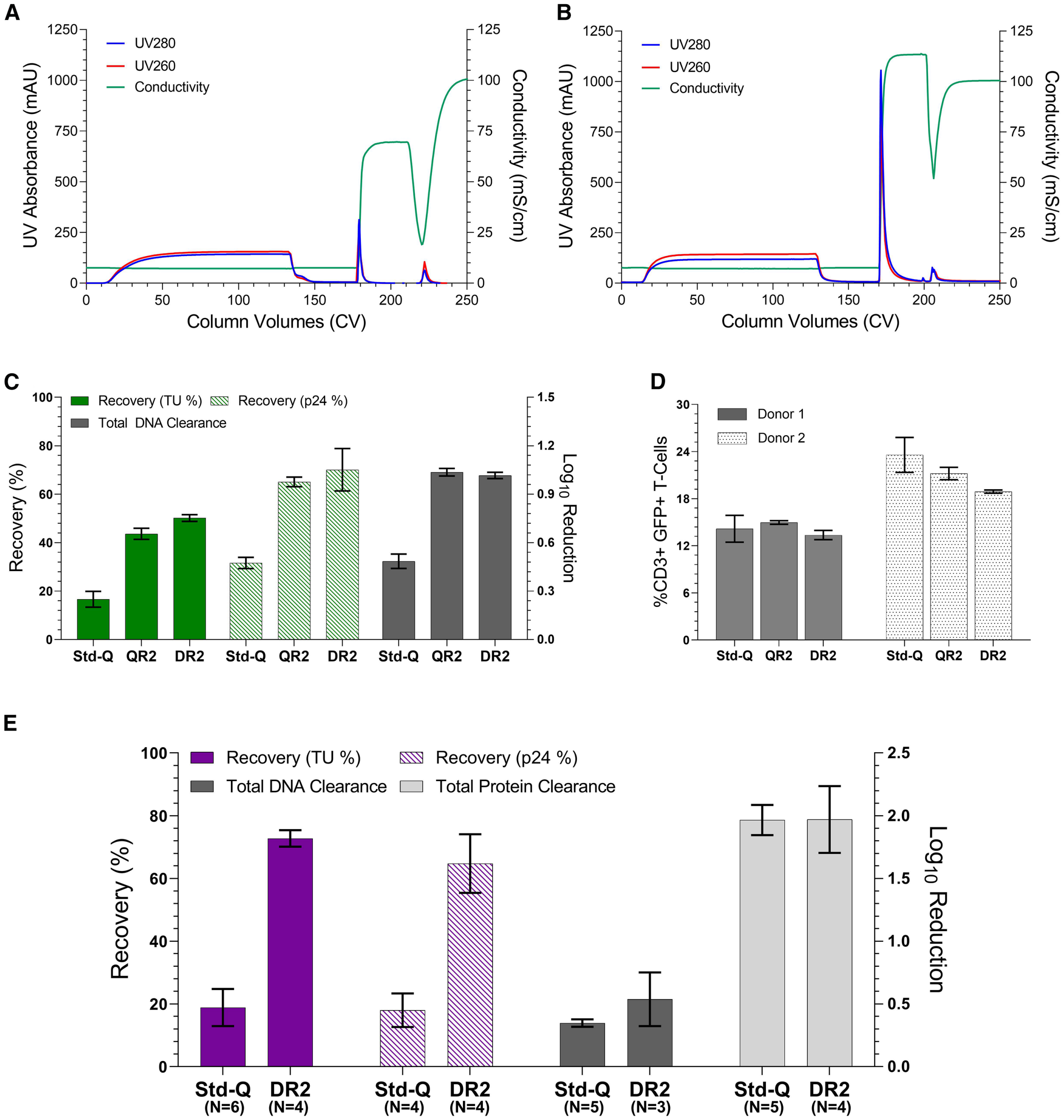

Figure 6. Purification performance of DR2 and QR2 prototypes compared with the standard Q-membrane for LVs encoding a GFP or CAR transgene

Error bars represent ± 1SD for $N = 3$ biological replicates unless otherwise stated. (A) Representative chromatogram for QR2. Load, elution peak, and NaOH peaks are visible. (B) Representative chromatogram for a standard Q-membrane. (C) LV-GFP step elution performance. Functional titer (TU) recovery, total particle (p24) recovery, and total DNA log_{10} reduction are given. (D) T cell transduction efficiency of the LV-GFP step elution eluate. Cells were seeded from two donors and transduced at an MOI of 0.5. No significant difference is observed for either the QR2 (p value = 0.46 and 0.16) or DR2 (p value = 0.49 and 0.067) membranes compared to the Standard Q across both donors, respectively. (E) Purification of LV-CAR using standard Q and DR2 membranes. The load material was not diluted for the standard Q-membranes to provide a more representative load conductivity. Functional and total particle recovery are given alongside the clearance of total DNA and total protein. Error bars represent ± 1SD with biological replicates given on figure.

with 750 mM NaCl (representative chromatogram in Figures 6A and 6B).

Purification using the QR2 and DR2 membranes resulted in a significant increase in functional titer recovery when compared against the standard Q-membrane (17%), achieving average functional titer recoveries of 44% and 50%, respectively (Figure 6C). This trend was mirrored in the total particle recovery (32%, 65%, and 70% for standard Q-membranes, QR2 and DR2, respectively). In terms of functional titer recovery, this corresponds to a 2.6- and 3.0-fold increase

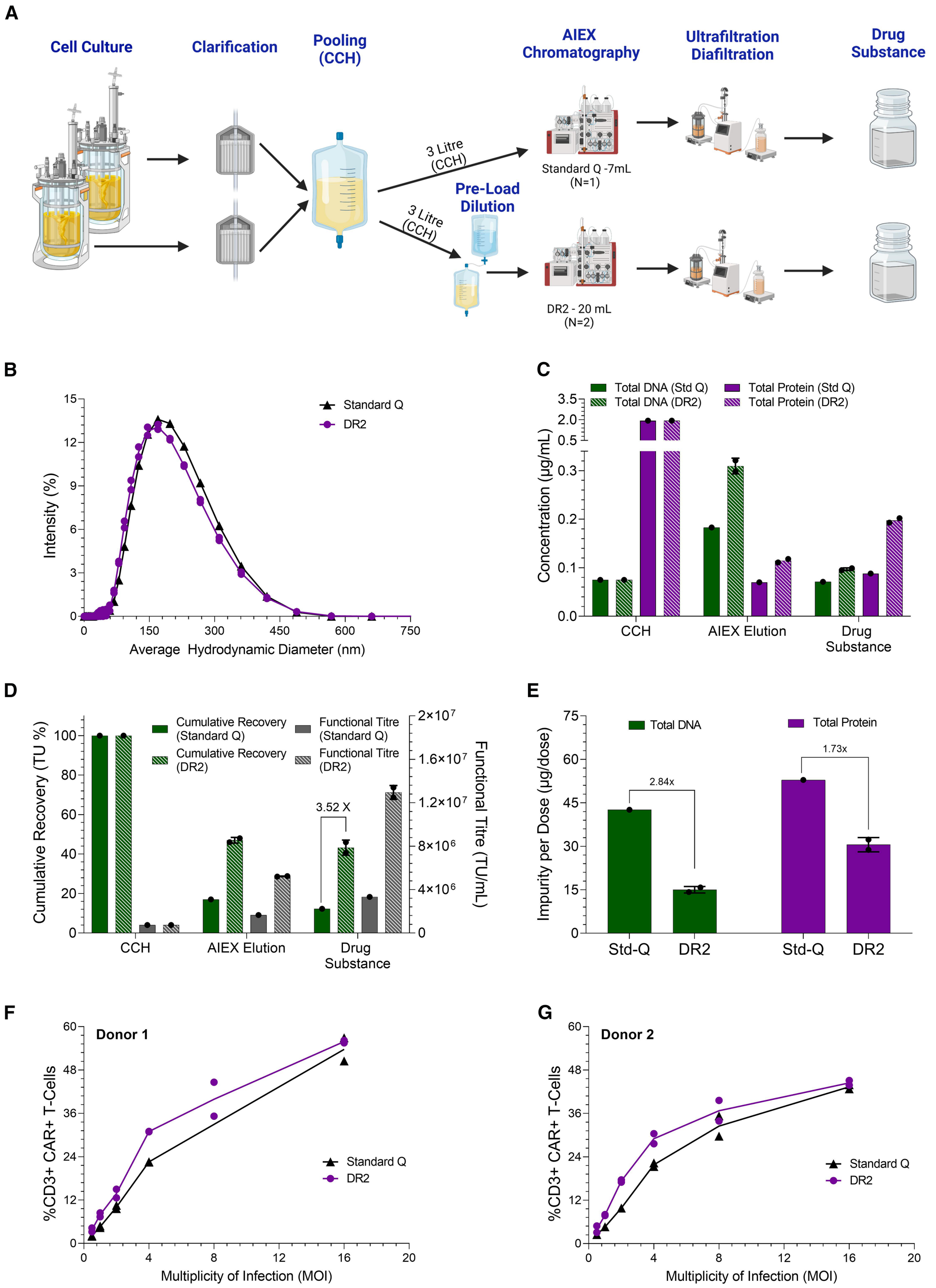

(legend on next page)

(legend on next page)

in eluted vector. Although only a small 6% increase in TU recovery was observed for the D chemistry compared to the Q, this was deemed significant at the $\alpha = 0.05$ level (p value = 0.02). No notable change was observed in the relative activity (22–25 TU/pg p24) of eluted vector (data not shown), which is expected given the reduction in recovery for standard Q-membranes was shown to result from physical retention of particles and not de-activation of eluted vector. A substantial increase in DNA removal was achieved with a $\log_{10}$ reduction of 1.04 (QR2) and 1.02 (DR2), respectively, compared to only 0.48 for the standard Q-membrane. This is likely a result of not only the tighter elution profiles of the prototypes meaning higher vector selectivity is achieved but also the overall lower binding of residual species as shown in Figure 4G.

To see whether the eluted vector from each membrane differed in primary T cell transduction efficiency, eluate from each was used to transduce T cells from two donors at an MOI of 0.5 (Figure 6D). No significant difference in the percentage of CD3+ GFP+ cells was measured between the standard Q-membrane and either prototype across both donors. This indicates that eluates from both membrane prototypes transduce T cells with the same efficiency. Ergo, improved product recovery from the QR2 and DR2 membranes is likely to directly translate to increased quantities of active drug product.

Chromatographic purification of LV-CAR using the DR2 membrane

Previously only research-scale devices have been used for evaluation. To more accurately assess real process performance of the new membrane class, LV expressing a therapeutic CAR transgene (LV-CAR) was purified using bench scale 3 mL radial flow device formats manufactured by Sartorius. Due to restrictions in device manufacture, only the most promising prototype candidate was selected. The DR2 prototype was chosen as it previously displayed a significant increase in functional titer recovery from step elutions compared to the QR2 and had the best performance in terms of pressure build and elution peak homogeneity. Despite a clear increase in both functional titer recovery and DNA removal, a comparison of identical purification protocols for the standard Q-membrane and the DR2 could be considered biased as these membranes have inherently different characteristics. For example, the higher elution salt concentration required for standard Q-membranes enables a higher load conductivity ($\sim$ 150 mM NaCl), which in turn facilitates the generation of cleaner eluate by reduced residual binding. Thus, for this study a more representative purification protocol for standard Q-membranes was employed based on our previous work, loading 400 CV of CCH directly (no dilution, conductivity ∽13 mS/cm) to a standard Q-membrane followed by a 150 mM NaCl flush and 1,200 mM NaCl elution. In contrast, the DR2 was loaded with 217 CV of diluted CCH based on a DBC with this material (data not shown, conductivity ∽7 mS/cm), followed by a 50 mM NaCl flush and 750 mM NaCl elution.

Improvements in both functional and total particle recovery using the DR2 membrane were even more pronounced for the LV-CAR than those seen for the LV-GFP. The standard Q-membrane achieved functional and total particle recoveries of 19% and 18%, respectively (Figure 6E), typical of this membrane type.[13,18] In contrast, the DR2 membrane obtained functional and total particle recoveries of 73% and 65%, respectively (>3.5-fold increase). A minor increase in DNA clearance was shown for DR2 (0.54 $\log_{10}$ reduction) compared to standard Q (0.35 $\log_{10}$ reduction) but was deemed insignificant (p-value = 0.26). An equivalent clearance of total protein was achieved for both the DR2 and standard Q-membranes (1.97 $\log_{10}$ reduction for both). These data demonstrate that vector recovery improvements remain high with the DR2 prototype under representative purification conditions, yet similar residual clearances are observed likely due to the higher load conductivities used for standard Q-membranes reducing binding of contaminant species.

Performance of DR2 membrane in a large-scale downstream process producing LV-CAR drug substance

To complete the analysis of this new membrane, the performance of DR2 in a large-scale downstream process producing LV drug substance was assessed. Three liters of LV-CAR CCH were used per individual run. Two 7-L stirred tank reactors (STRs) were independently clarified, then pooled to produce the CCH (Figure 7A). Each run was then purified using either a 20 mL DR2 membrane device (N = 2) or a 7 mL standard Q-membrane (N = 1). Material loaded to DR2 devices was diluted 1:1 in 20 mM Tris buffer prior to load. Eluates from each AIEX run were carried on to ultrafiltration/diafiltration (UF/DF) to produce the LV drug substance. The same overall (CCH drug substance) 30$\times$ volumetric concentration factor was employed to give the same end volume. Based on the elution profiles shown in Figure 4, a reduced NaCl elution concentration of 500 mM was used for the DR2 to reduce co-elution of impurities, whereas the standard Q-membrane required a concentration of 1,200 mM NaCl. DR2 AIEX eluate was not diluted in this study.

A 2.8-fold improvement in AIEX step recovery was achieved for the DR2 (47% TU) when compared against the standard Q-membrane

Figure 7. Comparison of large-scale purification processes, for production of LV-CAR drug substance, using DR2 (*N* = 2) and a standard Q-membrane (*N* = 1)
Biological replicates are plotted individually unless otherwise stated. (A) Process flow diagram for each of the standard Q and DR2 membrane processes. (B) Particle size distributions of the drug substance material. (C) Residual concentrations at each process stage. (D) Functional titer and cumulative functional recovery at each process stage. Recovery starts at 100% at CCH. (E) Expected mass of total DNA and total protein per LV drug substance dose. An assumption of 2×10^9 TU/dose was made based on previous work.[14] Note, these values do not represent final drug product doses but are used as a process comparison tool. (F) Impact of LV drug substance MOI on percentage of Donor 1 CD3+ T cells expressing the therapeutic CAR transgene. Technical replicates are indicated on figure. (G) Impact of LV drug substance MOI on percentage of Donor 2 CD3+ T cells expressing the therapeutic CAR transgene. Technical replicates are indicated on figure.

(17% TU), Figure 7D. The subsequent UF/DF unit operation resulted in high functional step recoveries of 92% (DR2) and 72% (standard Q). This led to a 3.5-fold improvement in recovery to drug substance for the DR2 (43%) compared to the standard Q-membrane (12%). To see whether the material produced via each process differed in primary T cell transduction efficiency, we performed a titration using drug substance from each process at increasing MOIs to ascertain whether any difference in profile or saturation point occurred. No notable difference was observed between the two processes across two T cell donors (Figures 7F and 7G), indicating that on a per vector per cell basis each drug substance was equally efficient at transducing patients' primary T cells. These data demonstrate that the 3.5-fold gains made in functional TU recovery result in the same increase in efficacious product. We also performed particle size measurements using dynamic light scattering to ascertain differences in average particle size of the two drug substance samples with no notable difference observed (Figure 7B).

Despite using the same volumetric concentration factor, the DR2 membrane showed higher concentrations of major residual species (Figure 7C) in the drug substance (total DNA = 0.097 μg/mL, total protein = 0.198 μg/mL) compared to the standard Q-membrane (total DNA = 0.071 μg/mL, total protein = 0.088 μg/mL). However, the increased yield of the DR2 process allows for more doses from the same volume, reducing residuals per dose. Assuming 2×10^9 TU/dose,[14] Figure 7E shows the relative impurity mass per LV dose. The DR2 process achieves a 2.8-fold reduction in DNA mass per dose and 1.7-fold reduction for total protein. Thus, despite higher impurity concentrations, the DR2 process results in lower residual species per LV dose. Note that these values are for process comparison and do not represent real administered values, as further processing occurs to generate final formulated LV drug product. It is also worth noting that despite impurity differences, both materials performed as efficiently during T cell transduction (Figures 7F and 7G). These data highlight the significant improvement to both LV recovery and purity achieved in devices scalable to GMP production sizes, achieving 3.5 times more LV-CAR doses per batch when compared against current membrane-based manufacturing processes.

DISCUSSION

This work demonstrates that time-dependent product loss of LVs on current commercially available standard Q-membranes can be mitigated by increasing membrane occupancy (bound mass). Through contact time experiments using increasing load volumes (Figure 1), we observed that increasing membrane occupancy led to a notable improvement in LV recovery, with the recovery halving-time ($t_{Y_{1/2}}$) increasing from 2.69 min (40 CV) to 7.14 min (200 CV). Despite achieving ∼60% recovery at short contact times, the high flow rates required for this approach present challenges for large-scale implementation, limiting its practicality. As we hypothesized that the interaction strength between LV and the membrane was driving these time-dependent losses, a new membrane material was designed to address this issue. Three critical adsorbent features were modified to reduce LV-membrane interaction strength: ligand density, ligand attachment method, and pore size (Figure 2). The ligand density was reduced from 100 to 8 μmol/mL, and the ligands were attached directly to the membrane surface, instead of within a polymer-grafted hydrogel. A reduced and more uniform pore size distribution was used, changing from 3–5 μm (standard Q-membrane) to ∼1.05–1.32 μm, to mitigate capacity loss through increased ligand accessibility. Four prototype membranes were generated (Figure 2) with these characteristics, utilizing either quaternary amine (Q) or dimethylamine (D) anion-exchange chemistries, with two pore size distributions: narrow (R1, 1.05 ± 0.04 μm) and wide (R2, 1.32 ± 0.02 μm).

Despite prototypes having approximately 2-fold lower DBCs than standard Q-membranes (Figure 3), a substantial improvement in loss of LV with time spent in the adsorbed state occurred, with high total particle recoveries of 73%–92% achieved at 4 min spent in the adsorbed state (Figure 5). Importantly, this improved recovery appears largely independent of the time the LV remained in the adsorbed state, achieving 72%–85% at 100 min in the adsorbed state. These results affirm our hypothesis that strong interaction strengths are the primary driver of time-dependent LV loss in standard Q-membranes. Considering the relationship between adsorbent ionic capacity and time-dependent loss, which has also been reported in other vector systems, we recommend future designs for LV AIEX adsorbents focus on reducing interaction strengths to maximize recovery and view membrane capacity as a secondary consideration.[24] While costly affinity resins, such as Protein A, drive up overall production costs of monoclonal antibodies, the cost of AIEX membrane materials is minimal compared to that of lost product. This shifts the focus toward AIEX recovery, a major determinant of the cost per dose of LV drug substance.[14]

In addition to the improvements in LV recovery, we observed significant differences in the gradient elution profiles between the prototype membranes and the standard Q-membrane (Figure 4). The prototypes produced much tighter elution peaks at lower NaCl concentrations (50–450 mM) compared to the broader peaks seen with standard Q-membranes (150–1,350 mM) (Figure 4). Differences between the Q and D chemistries were also apparent. While Q-chemistry membranes still exhibited the "two-peak" behavior, the D-chemistry membranes produced a single, shouldered peak (Figures 4D and 4F). These peaks are thought to arise from discrete LV subpopulations in the cell culture mix and are therefore also present in the D-chemistry profiles, suggesting superimposition (reduced peak separation) of the populations during elution.[19] Given previously reported differences in T cell activity between the two LV subpopulations,[19,23] future work could explore the separation of these populations, potentially favoring Q chemistries for superior peak separation. However, from a product selectivity standpoint, the D-chemistry membranes may be advantageous as their tighter elution profiles minimize impurity co-elution (Figures 4C–4G).

Functional titer recoveries for the prototype membranes were approximately 3-fold higher than those achieved with standard

Q-membranes across two different transgenes, with the DR2 membranes recovering 50% of LV-GFP and 73% of LV-CAR transducing units (Figure 6). Additionally, DNA removal was significantly better with the QR2 and DR2 membranes when using identical purification protocols. However, when using higher loading conductivities on the standard Q-membrane to reduce residual binding, this resulted in largely comparable residual removal between the DR2 and standard Q-membrane.

The best-performing membrane, DR2, was scaled up to 20 mL membrane capsules ($N = 2$) and used to purify 3 L of cell culture harvest. in an end-to-end downstream process (Figure 7). This process was compared to a similar process using standard Q-membranes to also purify 3 L of cell culture harvest. The DR2-based process achieved a 2.8-fold improvement in functional vector recovery at the anion-exchange chromatography step. Minimal product losses were observed for DR2 membranes during the subsequent ultrafiltration/diafiltration stage, resulting in a 3.5-fold improvement in functional product recovery at drug substance (DR2—43%, Standard Q—12%) with comparable T cell transduction efficiencies. Additionally, the DR2 process resulted in a 2.8-fold reduction in DNA mass per dose and a 1.7-fold reduction in total protein mass per dose.

These results underscore the significant improvement in LV purification that can be achieved by combining the beneficial properties of membranes for the capture of larger targets with binding properties specifically designed for reversible binding of LV, rather than adapting existing materials not designed for this purpose. We have demonstrated that targeted adsorbent redesign can eliminate key LV loss mechanisms, resulting in a 3-fold improvement in product recovery compared to currently available materials. We would have liked to understand which of the modified properties (i.e., pore size, ligand density, and hydrogel grafting) contributed most significantly to the observed improvements. However, our ability to perform an evaluation on this scale was constrained due to the interdependence of these parameters and difficulties associated with manufacturing the large number of prototypes required for this type of study. A way to enable these studies would be highly desirable but is beyond the scope of this paper as our primary goal was to develop commercially relevant materials capable of being manufactured at scale.

Our findings suggest that this next-generation chromatography membrane has the potential to significantly improve the productivity and robustness of LV downstream processing and has subsequently been commercialized under the trade name “Sartobind Convec” for industrial use. We recommend applying this tailored approach to other adsorbent morphologies and vector systems to achieve similarly high recoveries and robust processes. More broadly, treating viral vectors as a homogeneous product class that can be uniformly targeted using the same adsorbent materials greatly oversimplifies the diversity of viral vector structures and the intricate adsorption mechanisms they entail. We recommend that designers of chromatography materials focus on developing adsorbents specifically tailored to distinct viral vector separations. Such materials are essential for enabling manufacturers to meet the growing demand for viral products and enable the progression of cell and gene therapies to market.

MATERIALS AND METHODS

Cell culture and clarification

Third-generation HIV-1 LVs were generated following multi-plasmid co-transfection of the suspension-adapted HEK293T 1.65s cell line (Oxford Biomedica) as described in Pamenter et al.. Briefly, cells were inoculated at approximately 1×10^6 cells/mL in serum-free FreeStyle 293 Expression Media (Thermo Fisher Scientific) in glass STRs (Applikon) and agitated using an impellor stirring rate of 290 rpm. Cells were incubated at a temperature of 37°C, a pH set point of 7.2, and dissolved oxygen was maintained in excess of 20% throughout using an air/oxygen mix supplied via a sintered bead porous sparger. LV production was instigated via transient co-transfection of cells with four viral production plasmids: pOXB-GP (gag-pol protein and viral enzymatic components), pOXB-Rev, pOXB-VSV-G (envelope), and pOXB-GFP/CAR (transgenes). Approximately 24 h before vector harvest, LV production was stimulated by supplementation with the histone deacetylase inhibitor sodium butyrate (Sigma-Aldrich, Merck). At the termination of the production phase, the STR’s contents were clarified using a 0.2 μm normal flow filter, as previously described, to generate the CCH.[18] This was stored at −80°C before use in chromatography studies. For the large-scale study (Figure 7), material was not frozen, with the full downstream process completed immediately following bioreactor harvest. All LV-GFP materials originated from a single STR source. LV-CAR material was generated individually for each study.

Membrane characterization

All membrane materials were generated in house by Sartorius. Standard Q-membrane refers to the commercially available Sartobind Q anion-exchange membrane. Prototype membrane materials were generated using Sartorius’ proprietary technology. Four prototype materials were produced using Q and D chemistries at two pore sizes. Mean flow pore sizes were measured using a POROLUX 500 gas-liquid porometer and found to be 1.05 ± 0.04 μm (R1) and 1.32 ± 0.02 μm (R2). Thus, the four prototypes are as follows: QR1 (Q chemistry, 1.05 μm pore), QR2 (Q chemistry, 1.32 μm pore), DR1 (D chemistry, 1.05 μm pore), and DR2 (D chemistry, 1.32 μm pore).

Ligand densities were determined by loading the membrane samples with an excess of aqueous 0.1 M potassium phosphate buffer (pH 7.0). Excessive salt was removed by rinsing with deionized water. Immobilized phosphate was eluted with an aqueous solution of 10% (w/w) Na_2SO_4. Aliquots of the eluate were mixed with an equal amount of testing solution (0.5% [w/w] ammonium heptamolybdate tetrahydrate, 10% [w/w] sulfuric acid, and 2% [w/w] L(+)-ascorbic acid in deionized water). The mixture was incubated at 65°C to 75°C for at least 15 min. The phosphate concentration was determined photometrically at 825 nm using a phosphate standard as a reference.

For confocal imaging, membrane prototype samples were rinsed in deionized water and ethanol and dried at 80°C, followed by an

incubation in an ethanolic solution of AF647 (10 μg/mL). Excessive dye was removed by rinsing in ethanol, and the stained membrane sample was dried at 80°C. Samples were soaked in glycerin and imaged on a Leica TCS SP8 confocal laser scanning microscope equipped with an HC PL AP0 63×/1.40–0.60 OIL objective using glycerin as an immersion medium. For Sartobind Q, membrane samples were rinsed in binding buffer (20 mM Tris-HCl buffer [pH 7.2]), followed by incubation in an AF647 solution (10 μg/mL) in binding buffer. Excessive dye was removed by washing in binding buffer. Images were acquired using an HC PL AP0 63×/1.20 W CORR CS2 water immersion objective using otherwise identical procedures.

Anion-exchange chromatography

CCH, initially frozen at −80°C, was rapidly thawed in a 37°C water bath before experimental runs. All experiments were conducted using an ÄKTA Avant 150 chromatography system (Cytiva). Before processing, ÄKTA systems and membranes units were subject to a decontamination in 0.5 M NaOH for 1 h. Membranes were then charged with 2,000 mM NaCl and equilibrated in 50 mM NaCl. Membranes of varying volumes—0.08 mL or 0.345 mL (research scale), 1 or 3 mL (bench scale), and 7 or 20 mL (large scale)—were utilized based on the experimental protocol and material availability. Three process buffers, all formulated with 20 mM Tris with different NaCl concentrations, were mixed inline using the ÄKTA system to achieve specific salt gradients or isocratic elution concentrations. Specifically, the buffers were dilution buffer D (0 mM NaCl), buffer A (50 mM NaCl), and buffer B (2,000 mM NaCl). All CCH was diluted 1:1 using the dilution buffer prior to loading onto the ÄKTA system, unless otherwise stated, to achieve a suitable loading conductivity. Volumes in CVs are quoted based on pre-dilution volumes, unless otherwise stated.

For the chromatography membranes, all small-scale research devices (0.345 mL) utilized three-layer research units. For radial flow devices (≥1 mL), Sartobind Q membranes utilized 4 mm bed height, and prototype devices utilized 8mm bed height.

Contact time experiments

To generate kinetic profiles of LV recovery loss over time in the adsorbed state, we followed our previously described protocol.[18] One milliliter Sartobind Q Nano membranes were loaded with 40, 120, and 200 CV of CCH at 65 CV/min. After loading, a 30 CV wash with 150 mM NaCl at 65 CV/min flushed CCH from the system hold-up, and elution with 15 CV of 1,350 mM NaCl was performed. The eluate was collected in 45 mL of buffer D, resulting in an immediate 4-fold dilution to 300 mM NaCl. This procedure provided an average adsorbed contact time of 6.61 min for the 200 CV load. Extended time points were generated by "on-column" static incubation (e.g., 35-min contact time was achieved using a 28.39-min incubation). Incubations were performed after the 150 mM NaCl flush, as indicated in Figure 1C.

For the calculation of recovery in contact time experiments, specifically for generating kinetic loss profiles, only the recovery of bound vector material is considered. Accordingly, recovery is calculated based on the bound mass, determined by subtracting the flow-through mass from the initial loaded mass, as previously described in Pamenter et al.

Dynamic binding capacity

Membranes of volume 0.08 mL (Sartobind Q) and 0.345 mL (Prototype) were loaded with 810 CV (1,620 CV post dilution) of CCH diluted 1:1 in buffer D at a flowrate of 10 CV/min. Samples of the flowthrough were taken at 22 CV intervals and measured for p24 concentration.

Linear gradient elutions

Membranes of volume 0.08 mL (Sartobind Q) and 0.345 mL (Prototype) were loaded with 58 CV (116 CV diluted) of LV-GFP CCH at 10 CV/min. After loading, columns were flushed with 50 mM NaCl buffer for 40 CV. An elution range of 50–1,350 mM NaCl (Sartobind Q) and 50–750 mM NaCl (prototypes) was conducted by mixing buffers A and B. The same gradient slope of 6.1 mM/CV was applied to each membrane giving gradient length of 213 CV (Sartobind Q) and 115 CV (prototypes) to achieve the same resolution. Samples were fractionated every 2.9 CV.

Isocratic elution of LV-GFP and LV-CAR

For direct step elution comparison using LV-GFP, 1 mL (Sartobind Q) and 0.345 mL (Prototypes) membranes were loaded with 58 CV of diluted LV-GFP CCH at 10 CV/min, then flushed with 40 CV of buffer A. Elution was conducted using 30 CV of elution buffer, 1,200 mM NaCl for Sartobind Q, and 750 mM NaCl for the prototypes. The eluates were immediately diluted in buffer D to a final concentration of 200–250 mM to prevent salt-related infectivity loss that could affect functional titer assay readouts. All elutions were followed by a 0.5 M NaOH strip to remove any strongly bound material from the membrane.

For purification of LV-CAR an elution protocol more representative to real processing conditions was used. As the higher concentration of salt required to elute LV from Sartobind Q enables a higher load conductivity (150 mM NaCl), which also prevents the binding of residual species, adaptations to the loading condition were applied. A Sartobind Q membrane was loaded with ∼400 CV of un-diluted CCH based on our previous DBC work with this membrane and load material (data not shown). For the prototypes, 3 mL bench-scale devices were loaded with 220 CV (440 CV post dilution) of clarified CCH as determined by DBC (data not shown). The subsequent chromatography procedure was then the same as given above for LV-GFP.

Large-scale full downstream process of LV-CAR

LV-CAR cell culture was conducted as described previously. Two STR sources were used, which were independently clarified before both filtrates were pooled to generate the starting CCH material. For standard Q (Sartobind Q), 3 L of CCH was loaded directly to a 7 mL membrane device (429 CV) at a flowrate of 4 CV/min. This was followed by a 150 mM NaCl post-load flush. Elution was conducted at 1,200 mM

NaCl. The eluate was diluted 1:1 in buffer D to give a final product pool concentration of 600 mM NaCl. For each of the two DR2 runs, 3 L (150 CV) of CCH was diluted 1:1 prior to loading. This was followed by a 50 mM NaCl post load flush. Elution was conducted at 500 mM NaCl, and the eluate was not diluted further.

All eluate was loaded onto a 500 kDa hollow fiber (Cytiva). Due to differences in eluate volume from AIEX, all elution fractions (DR2 and standard Q) were concentrated at different concentration factors in order to achieve the same overall volumetric concentration factor of 30 from CCH to drug substance. Material was then diafiltered with 10 diavolumes of 100 mM NaCl and 20 mM Tris-based buffer to generate drug substance.

p24 concentration and functional vector measurement

The titer and recovery of LV after each unit operation is often measured in terms of both total physical particles, as measured by p24 concentration, and functional particles. The concentration of HIV-1 p24 was determined using the Ella (Protein Simple) high-throughput automated HIV-1 p24 ELISA.

Functional particle concentration was assessed using the method described in Pamenter et al.. In summary, samples were diluted with Dulbecco's modified Eagle's medium containing polybrene and added to adherent HEK293T cells in duplicate or triplicate, depending on the assay size. Cell analysis was conducted with an Attune NxT acoustic focusing flow cytometer (Thermo Fisher Scientific), measuring cell size and transgene fluorescence to identify the percentage of cells surpassing the background fluorescence of non-transduced cells. Functional titer was then calculated using Equation 1:

$$Titre\left(\frac{TU}{ml}\right) = \frac{[\%\ cells\ expressing\ Transgene \times number\ of\ cells\ at\ transduction \times dilution\ factor]}{Volume\ of\ Vector\ added\ at\ transduction} \quad \text{(Equation 1)}$$

Relative activity is determined by the functional titer to p24 ratio, Equation 2:

$$Relative\ Activity\left(\frac{TU}{pg}\right) = \frac{Functional\ titre\left(\frac{TU}{mL}\right)}{p24\left(\frac{pg}{mL}\right)} \quad \text{(Equation 2)}$$

Particle size measurement

Particle size distributions were measured using a Zetasizer Ultra (Malvern Scientific) dynamic light scattering machine. Measurements were performed in triplicate and analyzed 100 μL of sample at 20°C using a 633 nm laser and detecting backscattered light at an angle of 173°.

Statistics, non-linear regression, and model fitting

All statistical analysis (regression and hypothesis testing) was conducted using JMP 18 statistical software (SAS). Significance is given at the $\alpha = 0.05$ level unless otherwise stated. Non-linear regression was performed using the "specialized modeling" platform, "non-linear." An empirical model was fitted to the isocratic elution kinetic data to characterize the loss curves. A full description of this modeling methodology is given in Pamenter et al. and is described by Equation 3'[18]:

$$Y = \frac{Y_0 - Y_\infty}{1+(Y_0 - Y_\infty)k_2 t} + Y_\infty \text{ where } t_{Y_{1/2}} = \frac{1}{(Y_0 - Y_\infty)k_2} \quad \text{(Equation 3)}$$

where Y = recovery, Y_∞ = recovery as $t \to \infty$, Y_0 = recovery at $t = 0$, k_2 = decay rate constant (min^{-1}), t = time (min), and $t_{Y_{1/2}}$ = the recovery halving-time.

DATA AVAILABILITY

Data are available upon reasonable request to Dr. Lee Davies (corresponding author).

ACKNOWLEDGMENTS

This research is conducted in partnership with and receives support from Oxford Biomedica (UK) Ltd. We also acknowledge the collaborative efforts and technical contributions of Oxford Biomedica (UK) research team members, with particular thanks to Mr. Nathan Franklin and Dr. Thomas Williams. Our appreciation extends to the Oxford Biomedica (UK) Process Research & Development Analytics Team for their work in developing and validating the Ella high-throughput automated HIV-1 p24 ELISA, which was instrumental in this study. Special thanks are due to Miss Donna Byrne. The following employees of Sartorius Stedim Biotech GmbH are also thankfully acknowledged: Dr. Michael Metze for porometry measurements, Mr. Tony Schreiber for technical assistance in chemical membrane modification and characterization, and Mr. Pascal Sandmüller for technical assistance in CLSM sample preparation and image acquisition.

AUTHOR CONTRIBUTIONS

Purification studies were conceived by G.P., D.G.B., and L.D. Membrane design was conceived and executed by A.K., F.T., and V.T. Membrane material characterization studies were executed by A.K. G.P. designed small-scale purification experiments (Figures 1, 2, 3, 4, 5, and 6D). G.P., C.L., and D.H.R. executed small-scale studies. Large-scale experiments were designed and executed by M.K., C.L., K.P., R.S., O.G., G.P., D.H.R., and L.D. A.K. designed and performed T cell transduction studies. Research was performed in the laboratory of K.M. and C.K. G.P., D.H.R., D.G.B., and L.D. wrote the manuscript with contributions from all other authors.

DECLARATION OF INTERESTS

D.H.R., C.L., M.K., K.P., R.S., O.G., C.K., K.M., and L.D. were all employees of Oxford Biomedica (UK) Limited at the time the research was conducted. None of the authors or their employer received any additional renumeration for this work. A.K., F.T., and V.T. were all employees of Sartorius Stedim Biotech GmbH at the time the research was conducted. Sartorius Stedim Biotech GmbH has a patent pending for the technology of the

membrane prototypes presented and sells respective chromatography consumables under the trade name "Sartobind Convec D."

SUPPLEMENTAL INFORMATION

Supplemental information can be found online at https://doi.org/10.1016/j.omtm.2025.101533.

REFERENCES

1. Bulcha, J.T., Wang, Y., Ma, H., Tai, P.W.L., and Gao, G. (2021). Viral vector platforms within the gene therapy landscape. Signal Transduct. Target. Ther. *6*, 53. https://doi.org/10.1038/s41392-021-00487-6.
2. Lewis, P., Hensel, M., and Emerman, M. (1992). Human immunodeficiency virus infection of cells arrested in the cell cycle. EMBO J. *11*, 3053–3058. https://doi.org/10.1002/j.1460-2075.1992.tb05376.x.
3. Kim, V.N., Mitrophanous, K., Kingsman, S.M., and Kingsman, A.J. (1998). Minimal Requirement for a Lentivirus Vector Based on Human Immunodeficiency Virus Type 1. J. Virol. *72*, 811–816.
4. Mátrai, J., Chuah, M.K., and Vandendriessche, T. (2010). Recent advances in lentiviral vector development and applications. Mol. Ther. *18*, 477–490. https://doi.org/10.1038/mt.2009.319.
5. Naldini, L., Blomer, U., Gallay, P., Ory, D., Mulligan, R., Gage, F.H., Verma, I.M., and Trono, D. (1996). In Vivo Gene Delivery and Stable Transduction of Nondividing Cells by a Lentiviral Vector. Science *272*, 263–267.
6. Milone, M.C., and O'Doherty, U. (2018). Clinical use of lentiviral vectors. Leukemia *32*, 1529–1541. https://doi.org/10.1038/s41375-018-0106-0.
7. FDA (2024). Approved Cellular and Gene Therapy Products. https://www.fda.gov/vaccines-blood-biologics/cellular-gene-therapy-products/approved-cellular-and-gene-therapy-products.
8. Cell and Gene Therapy Catapult (2023). The Cell and Gene Therapy Catapult UK clinical trials database. https://ct.catapult.org.uk/resources/clinical-trials-database.
9. King, S.R. (1994). HIV: Virology and Mechanisms of Disease. Ann. Emerg. Med. *24*, 443–449. https://doi.org/10.1016/S0196-0644(94)70181-4.
10. Nguyen, D.H., and Hildreth, J.E. (2000). Evidence for Budding of Human Immunodeficiency Virus Type 1 Selectively from Glycolipid-Enriched Membrane Lipid Rafts. J. Virol. *74*, 3264–3272.
11. Merten, O.W., Hebben, M., and Bovolenta, C. (2016). Production of lentiviral vectors. Mol. Ther. Methods Clin. Dev. *3*, 16017. https://doi.org/10.1038/mtm.2016.17.
12. Perry, C., and Rayat, A.C.M.E. (2021). Lentiviral vector bioprocessing. Viruses *13*, 268. https://doi.org/10.3390/v13020268.
13. Valkama, A.J., Oruetxebarria, I., Lipponen, E.M., Leinonen, H.M., Käyhty, P., Hynynen, H., Turkki, V., Malinen, J., Miinalainen, T., Heikura, T., et al. (2020). Development of Large-Scale Downstream Processing for Lentiviral Vectors. Mol. Ther. Methods Clin. Dev. *17*, 717–730. https://doi.org/10.1016/j.omtm.2020.03.025.
14. Comisel, R.M., Kara, B., Fiesser, F.H., and Farid, S.S. (2021). Lentiviral vector bioprocess economics for cell and gene therapy commercialization. Biochem. Eng. J. *167*, 107868. https://doi.org/10.1016/j.bej.2020.107868.
15. Ghosh, R., Koley, S., Gopal, S., Rodrigues, A.L., Dordick, J.S., and Cramer, S.M. (2022). Evaluation of lentiviral vector stability and development of ion exchange purification processes. Biotechnol. Prog. *38*, e3286. https://doi.org/10.1002/btpr.3286.
16. Orr, V., Zhong, L., Moo-Young, M., and Chou, C.P. (2013). Recent advances in bioprocessing application of membrane chromatography. Biotechnol. Adv. *31*, 450–465. https://doi.org/10.1016/j.biotechadv.2013.01.007.
17. Hardick, O., Dods, S., Stevens, B., and Bracewell, D.G. (2013). Nanofiber Adsorbents for High Productivity Downstream Processing. Biotechnol. Bioeng. *110*, 1119–1128. https://doi.org/10.1002/bit.24765/abstract.
18. Pamenter, G., Davies, L., Knevelman, C., Miskin, J., Mitrophanous, K., Dikicioglu, D., and Bracewell, D.G. (2023). Time-dependent sorption behavior of lentiviral vectors during anion-exchange chromatography. Biotechnol. Bioeng. *120*, 2269–2282. https://doi.org/10.1002/bit.28483.
19. Tinch, S., Szczur, K., Swaney, W., Reeves, L., and Witting, S.R. (2019). A scalable lentiviral vector production and purification method using mustang Q chromatography and tangential flow filtration. In Methods in Molecular Biology (Humana Press Inc), pp. 135–153. https://doi.org/10.1007/978-1-4939-9065-8_8.
20. Slepushkin, V., Chang, N., Cohen, R., Gan, Y., Jiang, B., Deausen, E., Berlinger, D., Binder, G., Andre, K., Humeau, L., et al. (2003). Large-scale Purification of a Lentiviral Vector by Size Exclusion Chromatography or Mustang Q Ion Exchange Capsule. BioProcessing J. *38*, e3286.
21. Pamenter, G., Davies, L., Lamont, C., Rahim, D., Knevelman, C., Miskin, J., Mitrophanous, K., Dikicioglu, D., and Bracewell, D.G. (2024). Lentiviral vector determinants of anion-exchange chromatography elution heterogeneity. Biotechnol. Bioeng. *121*, 2936–2951. https://doi.org/10.1002/bit.28766.
22. Nestola, P., Villain, L., Peixoto, C., Martins, D.L., Alves, P.M., Carrondo, M.J.T., and Mota, J.P.B. (2014). Impact of grafting on the design of new membrane adsorbers for adenovirus purification. J. Biotechnol. *181*, 1–11. https://doi.org/10.1016/j.jbiotec.2014.04.003.
23. Vicente, T., Fáber, R., Alves, P.M., Carrondo, M.J.T., and Mota, J.P.B. (2011). Impact of ligand density on the optimization of ion-exchange membrane chromatography for viral vector purification. Biotechnol. Bioeng. *108*, 1347–1359. https://doi.org/10.1002/bit.23058.
24. Turnbull, J., Wright, B., Green, N.K., Tarrant, R., Roberts, I., Hardick, O., and Bracewell, D.G. (2019). Adenovirus 5 recovery using nanofiber ion-exchange adsorbents. Biotechnol. Bioeng. *116*, 1698–1709. https://doi.org/10.1002/bit.26972.
25. Reichert, U., Linden, T., Belfort, G., Kula, M.-R., and Thömmes, J. (2002). Visualising protein adsorption to ion-exchange membranes by confocal microscopy. J. Memb. Sci. *199*, 161–166.
26. Diedrich, J., Heymann, W., Leweke, S., Hunt, S., Todd, R., Kunert, C., Johnson, W., and von Lieres, E. (2017). Multi-state steric mass action model and case study on complex high loading behavior of mAb on ion exchange tentacle resin. J. Chromatogr. A *1525*, 60–70. https://doi.org/10.1016/j.chroma.2017.09.039.
27. Yamada, K., McCarty, D.M., Madden, V.J., and Walsh, C.E. (2003). Lentivirus vector purification using anion exchange HPLC leads to improved gene transfer. Biotechniques *34*, 1074–1080. https://doi.org/10.2144/03345dd04.

OMTM, Volume *33*

Supplemental information

Membrane adsorber design

for lentiviral vector recovery

George Pamenter, Danyal H. Rahim, Ciaran Lamont, Maria Kapanidou, Kirstie Pemberton, Rui Sanches, Anurag Kulkarni, Oliver Goodyear, Carol Knevelman, Kyriacos Mitrophanous, Andre Krause, Florian Taft, Volkmar Thom, Daniel G. Bracewell, and Lee Davies

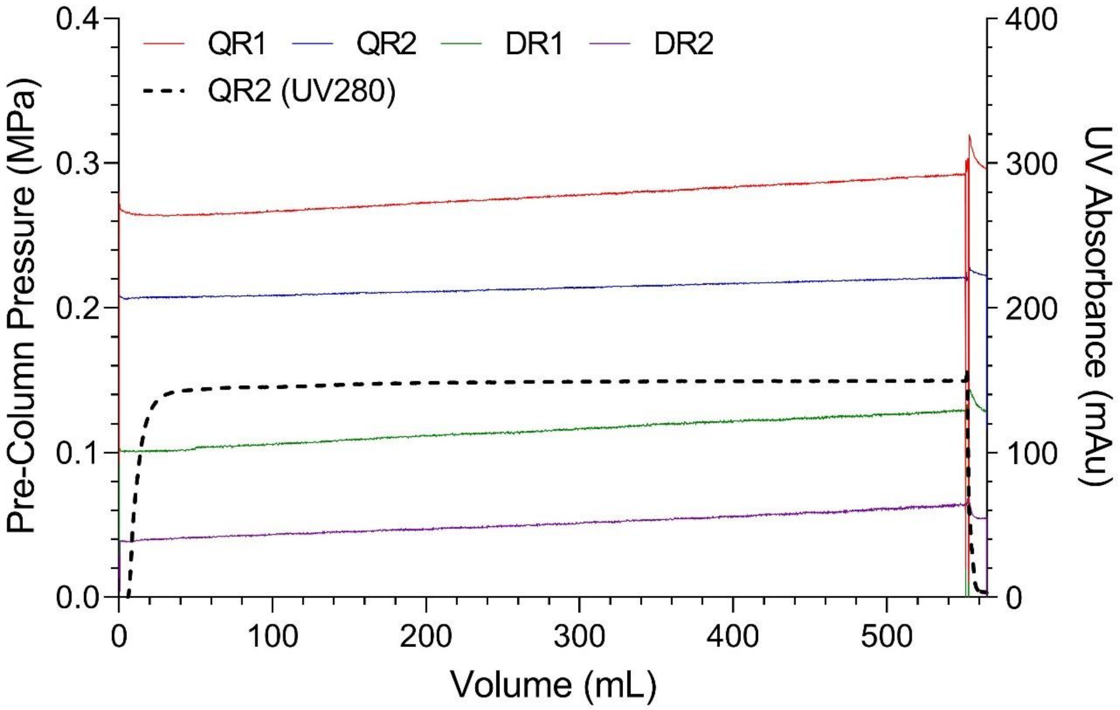

Figure S1: Pre-column pressure of the prototypes during dynamic binding capacity studies. Individual runs are given alongside the UV280 profile from a single run (QR2) to reference against for interpretation of the loading state.
